# Purslane Ameliorates Inflammation and Oxidative Stress in Diabetes Mellitus: A Systematic Review

**DOI:** 10.3390/ijms252212276

**Published:** 2024-11-15

**Authors:** Zikho Nkhumeleni, Wendy N. Phoswa, Kabelo Mokgalaboni

**Affiliations:** Department of Life and Consumer Sciences, College of Agriculture and Environmental Sciences, University of South Africa, Florida Campus, Roodepoort 1710, South Africa; 21098255@mylife.unisa.ac.za (Z.N.); mokgak@unisa.ac.za (K.M.)

**Keywords:** purslane, *Portulaca oleracea*, type 2 diabetes, inflammation, oxidative stress

## Abstract

Type 2 diabetes (T2D) is characterised by insulin resistance and leads to hyperglycaemia. Its prevalence and associated complications continue to rise exponentially, despite the existence of pharmaceutical drugs, and this has prompted research into exploring safer herbal remedies. *Portulaca oleracea* (purslane) has been investigated in animal and clinical trials to explore its effects on diabetes, yielding conflicting results. This study aimed to evaluate the effects of purslane on inflammation and oxidative stress in diabetes mellitus. We conducted a comprehensive literature search on Scopus PubMed, and through a manual bibliographical search to find relevant studies from inception to 13 September 2024. The search terms included purslane, *portulaca oleracea*, and type 2 diabetes mellitus. Of the 38 retrieved studies, 12 were considered relevant and underwent critical review. Evidence from rodent studies showed decreased inflammatory markers such as interleukin-6 (IL-6), tumour necrosis factor-alpha (TNF-α), nuclear factor kappa-beta (NF-κβ), and C-reactive (CRP), while interleukin-10 (IL-10) was increased after intervention with purslane. The markers of oxidative stress such as superoxide dismutase (SOD), catalase (CAT), glutathione (GSH), glutathione peroxidase (GPx), and total antioxidant capacity (TAC) levels increased, thiobarbituric acid reactive substances (TBARS), reactive oxygen species (ROS) and malondialdehyde (MDA) decreased. Notably, the evidence from clinical trials showed a significant reduction in NF-κβ and CRP after purslane treatment; however, no effect was observed on MDA and TAC. The evidence gathered in this study suggests that purslane exerts anti-inflammatory properties by downregulating NF-κβ, thus suppressing the production of associated pro-inflammatory cytokines. Therefore, purslane may be used as an antioxidant and inflammatory agent for diabetes. However, further clinical evidence with a broader population is required to validate the therapeutic properties of purslane in diabetes.

## 1. Introduction

Type 2 diabetes mellitus (T2D) is a metabolic disease characterised by insulin resistance and impaired glucose homeostasis with subsequent hyperglycaemia [1]. According to the World Health Organization (WHO) [2], T2D commonly occurs in adults, and the number of individuals living with it has risen exponentially over the past 30 years worldwide [2]. In South Africa, the prevalence of T2D increased from 4.5% in 2010 to 12.7% in 2019 [3]. The prevalence rate of diabetes was 12.2% globally in 2021 [4]. The rise in T2D can be attributed to obesity, an inactive lifestyle, high-calorie diets, and ageing [5,6]. Obesity induces insulin resistance by releasing high levels of fatty acid and impaired secretion of adipokines [5].

Previous research has shown that inflammation is an important contributor to the manifestation of T2D [6]. There is an increased inflammatory response in patients living with T2D, as demonstrated by elevated levels of inflammatory markers such as interleukin 6 (IL-6), tumour necrosis factor-alpha (TNF-α), and C-reactive protein (CRP) present in the blood [7]. This increases the risk of T2D-associated complications, such as macrovascular complications (diseases of the coronary arteries, peripheral arteries, and blood vessels of the brain) [8], and those of a microvascular nature, such as diabetic neuropathy, nephropathy, and retinopathy [9]. Therefore, controlling hyperglycemia in T2D can reduce the complications mentioned above.

Oxidative stress is a central feature of T2D, and is characterised by excess reactive oxygen species (ROS), which leads to insulin resistance [6,10]. The buildup of these ROS molecules and reactive nitrogen species (RNS) in mitochondria impairs its function, inducing insulin resistance and inflammation [5,10,11]. Furthermore, oxidative stress can damage the endothelial lining, resulting in reduced production of the nitric oxide necessary for vasodilation [5,12]. This subsequently results in impaired blood vessels, which become susceptible to atherosclerosis [5,13]. The latter contributes to an increased risk of cardiovascular disease (CVD) among patients living with T2D [8]. Therefore, it is important to control oxidative stress to ameliorate atherosclerosis and associated CVD among patients living with T2D.

**Figure 1:**
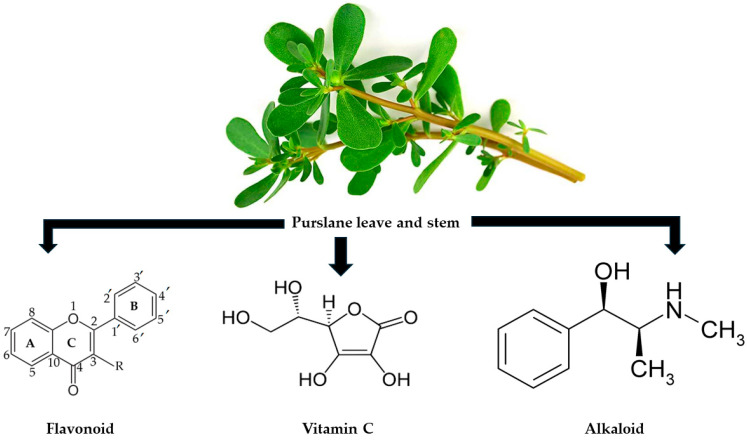
Purslane and its active compounds [26,29,31]. An active compound is found in purslane leaves and stems.

Although T2D patients rely on anti-diabetic drugs, such as Glucophage and sulfonylurea, to control hyperglycaemia, the secondary side effects and complications associated with their use seem to be intolerable in other patients [14,15]. These side effects include diabetic ketoacidosis, gastrointestinal events, and severe hypoglycaemia [15,16,17]. Therefore, the above-mentioned side effects warrant an urgent need for alternative therapies that are safe and tolerable. Several scientists globally have been researching alternative ways to treat T2D, especially herbal remedies [18,19]. It is worth noting that the WHO has also endorsed using herbal remedies in T2D due to their efficacy and safety profile [20]. Some of these herbs have some limitations; for instance, our team recently found that while *Abelmoschus esculentus* has benefits in reducing fasting blood glucose, it has no effect on glycated haemoglobin as one marker of hyperglycaemia [21]. However, *Portulaca oleracea,* known as purslane, has been studied in rodent models of T2D, and it has shown some potential benefits in reducing blood glucose and total cholesterol [22]. This natural herb is found as a weed that belongs to the purtulaceae family [23]. The herb is distributed globally [23] and is an excellent source of antioxidants, such as vitamins A and C, omega-3 fatty acids, and alpha-linolenic acid [23,24,25,26]. The phytochemicals in purslane include flavonoids, alkaloids, and terpenoids [27,28,29]; these enhance purslane antioxidant capabilities. These active compounds and nutrients contribute to the potential benefits of purslane, as observed in T2D. Notably, YouGou et al. 2009 reported the free radical scavenging properties of purslane due to the active compounds mentioned above [30]. Some of the important active compounds found in the leaves and stems of purslane, as presented in Figure 1, have significant health benefits. Therefore, the proposed study aims to review the effect purslane has on markers of inflammation and oxidative stress in diabetes mellitus.

## 2. Method

The study adhered to preferred reporting items for systematic review and meta-analysis (PRISMA) guidelines [32] (Supplementary File S1) and followed PICO criteria, which were outlined as follows: participants with type 2 diabetes mellitus and animal models of diabetes, purslane, and its extracts, healthy control group or placebo, markers of inflammation and oxidative stress. The protocol for this review was not registered; however, no duplicate study was found in the database.

## 3. Information Source and Search Strategy

PubMed and Scopus were used by independent scholars (ZN and KM) to conduct an extensive literature search. The following MeSH terms and Boolean operators were used: “type 2 diabetes” or “type 2 diabetes mellitus” and “purslane” OR “*Portulaca oleracea*”. No restrictions were applied in terms of study designs. The exact search adapted to the databases was prepared for records published from inception until 13 September 2024 (Table 1).

## 4. Eligibility Criteria and Selection Process

All studies that met the following PICO criteria were included in the study: the target population was adult (19+ years) patients living with T2D and diabetic rodent models, the intervention was purslane or purslane extracts as treatment, the control was diabetic patients or rodent models on standard treatment of T2D, and the outcomes of the study were the changes in inflammation and oxidative stress. All studies that reported no markers of inflammation or oxidative stress were excluded. Studies conducted in patients without diabetes or non-model of diabetes and studies not on purslane treatment were excluded. Reviews, non-English publications, abstracts, letters, and dissertations were also excluded.

## 5. Data Extraction

Two scholars (ZN and KM) independently extracted data from all relevant studies. The following data were extracted from each of the studies: the first author’s surname, the country where the study was performed, the study design, the duration of treatment with purslane, the dose, the form of purslane treatment, and the main findings. Any disagreement in terms of data extraction was resolved through discussion with a third independent scholar (WNP) who thoroughly reviewed the screened studies and extracted data before a final decision was taken.

## 6. Results

**Figure 2:**
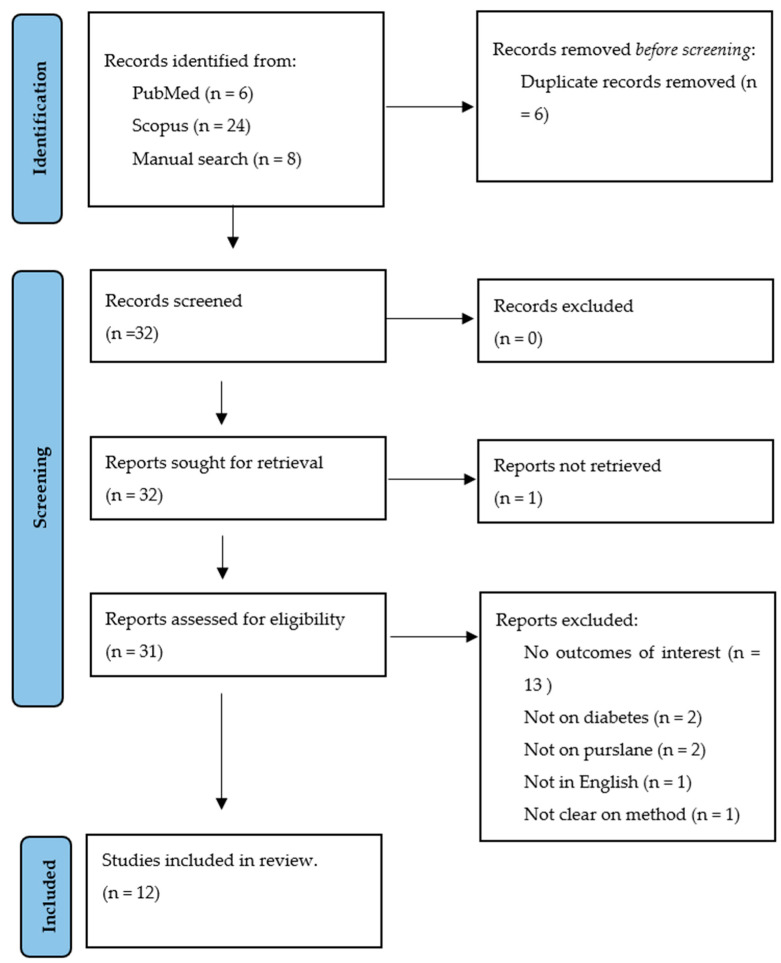
PRISMA flow chart depicting study selection, screening and inclusion.

A total of six records were obtained from PubMed, while 24 were obtained from Scopus and eight through a manual bibliography search. Before the screening, six records were found to be duplicates in the databases and were therefore excluded. This left 32 records to be screened based on their titles, abstracts, keywords and objectives. Of these 32, one could not be retrieved. Thirteen of the records were excluded because the outcomes were not relevant to this study, two were excluded because the intervention was not purslane, two were excluded due to it not being on T2D, one was excluded for not being written in English due to language barrier, and one was excluded because the methodology was not transparent. Thus, 12 articles were deemed relevant to the purpose of this study, 10 of which were rodent models of diabetes studies [22,33,34,35,36,37,38,39,40,41], while two were clinical trials on T2D [42,43] (Figure 2).

## 7. Characteristics of the Included Studies

We identified ten pre-clinical studies published in peer-reviewed journals between 2008 and 2024 relevant to our research. These studies were published mainly in Asian countries, including India [34,39], Iran [35], China [36], Iraq [22], and Korea [38] with the exceptions of Egypt [33,37,40] and Nigeria [41]. Different rodents were used, including rats (Sprague Dawley and Wistar Albino) and mice (C57BL/KsJ-db/db and C57BL/6J). Different methods were used to induce diabetes in these rodents, such as intraperitoneal injection with streptozotocin (STZ), alloxan monohydrate or high-fat diet (HFD) feeding (Table 2).

We also retrieved two clinical studies that investigated the effect of purslane on inflammation and oxidative stress in T2D patients. The studies were also published in peer-reviewed journals between 2015 and 2016. These studies were conducted in Iran [42,43]. A total of 236 diabetic patients were included in these studies. The purslane was administered orally as a capsule and seed powder mixed with low-fat yoghurt (Table 3). These two studies were a double-blind study and randomised cross-over clinical trials.

## 8. The Effect of Purslane and Its Derivatives on Markers of Inflammation in Rodent Models of Diabetes

Evidence from rodent model experiments has shown that purslane does have the potential to act as an anti-inflammatory treatment. For instance, Hassan et al. [33] used STZ-induced diabetes in adult male Sprague Dawley rats to investigate the effects of 100 mg/kg of ethanolic extract of purslane for 4 weeks. The diabetic rats treated with purslane showed a 38.6% decrease in TNF-α levels compared to the diabetic rats that received no treatment. This study also showed a 155% increase in IL-10 in the purslane-treated group.

Samarghandian et al. [35] used STZ-induced diabetes in Wistar albino rats, and these were treated with either 100 mg/kg, 200 mg/kg, or 400 mg/kg of purslane for 4 weeks. In this study, the 400 mg/kg dosage decreased serum IL-6 and TNF-α, with means of 3.45 ± 0.36 pg/mL, *p* < 0.05 and 10.26 ± 1.05 pg/mL, *p* < 0.01 respectively, compared to the untreated group (5.34 ± 0.54, *p* < 0.001 and 15.11 ± 1.13, *p* < 0.01). The 200 mg/kg dose decreased serum TNF-α, with a mean of 11.54 ± 0.49 pg/mL, *p* < 0.05 compared to the untreated group (15.11 ± 1.13, *p* < 0.01). Another study conducted in Egypt [37], showed that 250 mg/kg of purslane treatment in diabetic Wistar rats induced by alloxan monohydrate reduces TNF-α (35.2 ± 0.24 pg/mL), *p* ˂ 0.05 and IL-6 levels to 16.26 ± 0.17 pg/mL, *p* < 0.05. Additionally, Sook Lee et al. [38] treated C57BL/KsJ-db/db mice and male wild-type C57BL/6J mice with 300 mg/kg of aqueous purslane for 10 weeks. Here, the levels of NF-κβ p65 decreased significantly with *p* < 0.05. In a study by Saad et al. [40], STZ-induced Wistar rats were treated with 200 mg/kg of purslane seed extract for 30 days. Here, the levels of CRP, IL-6, and TNF-α dropped from +512.1%, +271.5%, and +236.6%, *p* < 0.0001, respectively, in the untreated group to −26.6%, −60.2%, and −42.7%, *p* < 0.0001, respectively, in the purslane-treated group. Similarly, Oyabambi et al. [41] treated STZ-induced Wistar rats with 400 mg/kg purslane for 4 weeks. This study revealed a significant decrease in TNF-α and IL-6 after treatment with *p* < 0.05.

## 9. The Effect of Purslane and Its Derivatives on Markers of Oxidative Stress in Rodent Models of Diabetes

Evidence from rodent model experiments has also shown a positive effect on oxidative stress following treatment with purslane. Briefly, in a model of diabetes induced by STZ in Spraque Dawley rats, treatment with purslane alone reduced levels of TBARS by 81.4%, *p* < 0.0001 and 58.7%, *p* < 0.001 [33]. This evidence shows that purslane is more effective in reducing TBARS than control. This study showed reduced hippocampal glutathione (GSH) levels and SOD activity after purslane treatment, *p* < 0.05. Similarly, in the STZ-induced diabetic model, there was an increase to normal levels of SOD (6.56 ± 0.02, *p* < 0.05, at 100 mg/kg, and 8.36 ± 0.22, *p* < 0.0001, at 250 mg/kg) in the liver following purslane treatment. Interestingly, the same observation was noted in the kidney SOD (4.34 ± 0.12, *p* < 0.05 at 100 mg/kg and 5.82 ± 0.10, *p* < 0.01, at 250 mg/kg) [34]. The same study reported a significant increase in CAT activity (47.82 ± 3.83, *p* < 0.01, at 100 mg/kg, and 60.71 ± 4.18, *p* < 0.001, at 250 mg/kg) in the liver. The same study revealed an increase in CAT (18.14 ± 1.74, *p* < 0.01, at 100 mg/kg, and 25.58 ± 2.21, *p* < 0.001, at 250 mg/kg) in the kidney following purslane treatment. The GSH levels increased in the liver and kidney (98.4 ± 6.71, *p* < 0.01, at 100 mg/kg, 118.9 ± 5.41, *p* < 0.001, at 250 mg/kg, 168.15 ± 3.35, *p* < 0.01, at 100 mg/kg, 94.70 ± 4.16, *p* < 0.001, at 250 mg/kg, respectively). Moreover, the STZ-induced Wister rats in Samarghandian et al. showed that 200 mg of purslane increased GSH levels (1.97 + 0.22, *p* < 0.01) and 400 mg of purslane increased GSH levels (2.36 + 0.15, *p* < 0.001) [35]. In the same study, 400 mg of purslane reduced MDA levels (2.47 + 0.29, *p* < 0.001) [35]. C57BL/6J mice and Sprague Dawley rats induced by an HFD feeding had reduced ROS levels in the aorta after purslane treatment, *p* < 0.05, compared to untreated diabetic rats [36]. Another study [22] that administered 200 mg/kg purslane in diabetes rats showed an increase in the activities of SOD and CAT (561.31 ± 27.51, *p* < 0.01, and 71.92 ± 3.38, *p* < 0.01, respectively). In a study by Sharma et al. [39], 25 mg/kg and 50 mg/kg of purslane reduced TBARS levels (3.10 ± 0.14, *p* < 0.01, at 25 mg/kg, and 2.12 ± 0.15, *p* < 0.001 at 50 mg/kg). The same study revealed an increase in GSH activity (99.54 ± 4.87, *p* < 0.01, at 25 mg/kg, and 127.24 ± 5.31, *p* < 0.001, at 50 mg/kg) and GPx activity (6.07± 0.54, *p* < 0.01 at 25 mg/kg, and 7.89 ± 0.64, *p* ˂ 0.01, at 50 mg/kg). Furthermore, there was an increase in SOD activity (58.13 ± 5.13, *p* < 0.01, at 25 mg/kg, and 74.51 ± 6.14, *p* < 0.001, at 50 mg/kg), and an increase in CAT activity (6.22 ± 1.84, *p* < 0.01, at 25 mg/kg, and 8.72 ± 2.44, *p* < 0.001, at 50 mg/kg). Saad et al. [40] reported a decrease in MDA levels from +37.3%, *p* < 0.0001 to −19.9%, *p* < 0.0001 after treatment with 200 mg/kg purslane. Additionally, there were significant increases in the levels of GSH (+115.1% and +136.1%, *p* < 0.0001), TAC (+82.3% and +78.6%, *p* < 0.0001), SOD activity (+27.3%, and +35.7%, *p* < 0.0001) and CAT activity (+27.3% and +27.4%, *p* < 0.0001). Oyabambi et al. [41] also observed a significant increase in GSH level, *p* < 0.05, compared to the untreated diabetic group.

## 10. The Effect of Purslane and Its Derivatives on Markers of Inflammation in Diabetic Patients

In this study, inflammation was assessed based on CRP and NF-kβ. The evidence reported by Dehghan et al. showed that purslane could decrease the expression of NF-κβ levels after 16 weeks of treatment (6.5 ± 0.4 ng/mL, *p* < 0.05) compared to pre-treatment (8.52 ± 0.5 ng/mL) [42]. Additionally, the same study also revealed a significant decrease in the level of the CRP in post-purslane treatment (6.2 ± 0.1 mg/mL) *p* < 0.05 compared to pre-treatment (7.85 ± 0.6 mg/mL).

## 11. The Effect of Purslane and Its Derivatives on Markers of Oxidative Stress in Diabetic Patients

The effect of purslane on oxidative stress was evaluated in one clinical trial [43]. The results of this study demonstrated an observable decrease in total antioxidant capacity (TAC) (10.5 ± 11.0 µg/dL from 3.2 ± 14.6 µg/dL). Still, the difference between baseline and post-purslane treatment was not significant, *p* = 0.15 [43]. This study also showed an observable increase in MDA levels between baseline and post-treatment (15.0 ± 13.2 µg/dL from 13.4 ± 15.2 µg/dL, *p* = 0.63).

## 12. Discussion

The global prevalence of diabetes in 2021 was reportedly 10.5% (536.6 million people) and is expected to rise to 12.2% (783.2 million people) by 2045 [4]. This suggests that the risk of developing cardiovascular complications associated with diabetes will also increase sharply in the coming years. Therefore, this study evaluated the effect of purslane as a natural herbal remedy in diabetes, focusing mainly on oxidative stress and inflammation. In this study, evidence from pre-clinical and clinical studies showed improved inflammation and oxidative stress in diabetes following treatment with purslane.

These findings support previous research done by Jafari et al. [44], which also shows a significant reduction in MDA and TAC after purslane therapy. However, our study differs as we see an increase in MDA in one of the clinical trials. Bai et al. [45] also reported consistent findings, where IL-6, TNF-α, and MDA were decreased, as well as an increase in SOD activity following purslane treatment. However, the clinical trial by Zakzadeh et al. [43] yielded contradicting results. Zheng et al. also obtained similar results, where a reduction in IL-6 and TNF-α during liver injury was observed in diabetic mice [46].

T2D patients have a high risk of oxidative stress [47], which is induced by an imbalance between ROS production and the body’s antioxidant capability, mainly in the mitochondrion [13]. This imbalance causes ROS to attack and alter functional and structural molecules, leading to tissue injury and dysfunction [48]. As a response to this tissue injury, inflammation begins [49], where immune cells secrete different cytokines and chemokines to recruit other immune cells to the site of oxidative stress [50]. This process triggers intracellular signaling pathways, including kinases and transcription factors (especially NF-κβ) [49]. Therefore, it is essential to control oxidative stress in diabetes to alleviate associated complications. The evidence presented in this study suggests that purslane may be used as an anti-oxidative and anti-inflammatory agent. As inflammation is known to induce adversity in T2D, including endothelial dysfunction [7], any potential approach that can ameliorate inflammation in diabetes can be used as a therapeutic target in the search for alternative potential treatment. The evidence gathered from animal models of diabetes has shown the potential of purslane to reduce inflammation by reducing pro-inflammatory cytokines. For instance, TNF-α [33,35,37] and IL-6 [35,37] were decreased in diabetic rats on purslane treatment compared to those without treatment. Interestingly, this was independent of the dosage. It is worth noting that TNF-α is a pro-inflammatory cytokine; thus, its elevation contributes to inflammation in T2D [51]. Additionally, TNF-α promotes the transcription of IL-6 by activating the NF-κβ pathway [52,53]. An active NF-κβ enters the nucleus, upregulating the expression of genes such as IL-6 and IL-1β [54,55]. An increased expression of such genes promotes their translation and production, which leads to inflammation. Interestingly, the results showed a reduced expression of NF-ĸβ following purslane intervention in the diabetes model [38]. Consistent with findings from animal studies, the clinical trial conducted by Dehghan et al. [42] also revealed a reduced NF-ĸβ in T2D after purslane treatment.

On the other hand, another trial that focused entirely on a different marker of inflammation, CRP, reported a reduction in CRP following 16 weeks of purslane treatment among T2D patients [42]. CRP can induce endothelial dysfunction by inhibiting the AMP-activated protein kinase endothelial nitric oxide synthase (AMPK-eNOS) signaling pathway [56]. AMPK activation suppresses NF-κβ activity, thereby reducing the expression of pro-inflammatory cytokines such as TNF-α and IL-6 [57]. Additionally, AMPK activation in the adipose tissue inhibits IL-1β-stimulated secretion of chemokine (C-X-C motif) ligand 10 (CXCL10). It reduces TNF-α-induced inflammatory signaling by downregulating the inhibitor of kappa-beta (Iκβ) and the Jun-N-terminal kinase (JNK) pathway [58,59]. AMPK can phosphorylate and inhibit Iκk, which is responsible for the phosphorylation and degradation of Iκβ proteins. It is worth noting that degraded Iκβ helps to move the NF-κβ to the nucleus and activates pro-inflammatory genes [57,58]. Purslane functions through this AMPK pathway to inhibit inflammation by suppressing NF-κβ expression and CRP production [60]. Oyabambi et al., 2024 have also shown that 400 mg/kg of ethanolic extract of purslane increased the expression of AMPK in diabetes rats [41]. Similarly, Miao et al. 2023 also reported an increased expression of AMPK following purslane treatment [36]. This potential effect of purslane on inflammation seems to be attributable to the content of active phytochemicals, such as the alkaloids and flavonoids found in purslane [61,62,63]. These findings support the potential effect of purslane as an anti-inflammatory herbal remedy for diabetes.

Anti-inflammatory cytokines also play a crucial role in regulating inflammation in diabetes [64]. One important anti-inflammatory marker evaluated in this study was IL-10. In this study, the level of IL-10 was increased following purslane treatment in diabetes. Indeed, IL-10 is an anti-inflammatory cytokine that inhibits the action of pro-inflammatory cytokines, including IL-2 [65]. The role of anti-inflammatory cytokines is to counteract the inflammatory cytokines that promote inflammation and complications thereof. In T2D, the level of IL-10 is reduced, thus predisposing this group of patients to inflammation and the associated complications [65]. However, purslane demonstrated its anti-inflammatory properties by significantly increasing its levels in rodent models of diabetes [33]. Purslane seems to alleviate inflammation by inhibiting the expression of NF-ĸβ, thus suppressing the expression and production of TNF-α and IL-6, which promotes the production of IL-10 [66,67]. Another study reports that purslane improves the function of immune cells, mainly the regulatory T-cells, which produce IL-10, thus promoting a shift towards anti-inflammatory cytokines [68,69]. This action allows for the production of more anti-inflammatory cytokines than pro-inflammatory cytokines.

Through evidence from rodent models, this review showed that purslane is a good source of antioxidant activity. Individuals living with T2D have been known to have decreased levels of the antioxidant enzyme GSH and GPx and increased SOD, which promotes oxidative stress as shown by increased ROS [70]. These antioxidant enzymes protect the cells from hydrogen peroxides that form as an end product of ROS in scavenging reactions [71]. In hyperglycaemic conditions, glucose uptake occurs in the polyol pathway, expanding the NADPH required for GSH production by the glutathione reductase (GR) enzyme [70]. A decrease in GPx is also expected in T2D due to the reduced content of GSH, as this is a substrate for GPx [70]. The findings from rodent models of diabetes showed an increase in levels of GSH and GPx following the administration of purslane, further confirming its antioxidant properties [33,34,35,39,40]. It is also important to note that increased oxygen free radicals and ROS production lead to lipid peroxidation [72]. These products of lipid peroxidation include MDA and TBARS [73]. This has reportedly contributed to atherosclerosis and microvascular and macrovascular complications associated with T2D [73,74]. The evidence from the rodent models [22,34,39] showed that the administration of purslane improved the activity of these enzymes. In rodent models, MDA was found to decrease post-purslane treatment [35], whereas the evidence from one clinical trial showed a slight increase in MDA levels after treatment [43]. These conflicts may be due to different mechanisms and absorption of purslane in rodents and humans. Individuals with T2D also have high levels of TBARS due to lipid peroxidation, which increases the risk of CVDs [75]. However, in this study, the TBARS level was reduced in rodent models of diabetes [33,34,39] after purslane treatment. Rani et al. demonstrated that patients with T2D have a decreased TAC associated with increased oxidative stress [76]. In a diabetic rodent model, we noted that purslane can ameliorate oxidative stress by increasing TAC [35]. This suggests that purslane can alleviate oxidative stress in T2D.

Similarly, we also observed a minor but not significant increase in TAC in T2D patients after treatment with purslane [43]. An increase in TAC in T2D thereby slowed down oxidative stress. While the potential benefits were observed in preclinical studies, the results still require validation in clinical trials on inflammation globally as the prevalence of diabetes is increasing gradually. Therefore, purslane can be used as a supplement to ameliorate oxidative stress in diabetes mellitus.

## 13. Strength and Limitations

Various limitations were noted in this review, such as the fact that the evidence reviewed in this study was limited to Asian countries, with one exception being one study in West Africa (Nigeria). While two human clinical trials were available, there was no variation in the demographics of patients, as both studies took place in Iran, and one only included female patients. We also noticed discordant results between rodent and human studies in the marker MDA. Although there were positive effects on oxidative stress in animal studies, the same findings were not observed in humans. This may be due to the dosage of purslane and the duration of the treatment, and may require further investigation. Different methods were used to induce diabetes in the rodents. However, this led to no significant differences in results. Different doses of purslane and intervention durations were used to treat the T2D patients, which may have led to conflicting results. Although the preclinical studies are useful in testing the efficacy and toxicity of the herbal therapy before clinical trials can be conducted, in some cases, the results are not fully replicable in clinical trials due to differences in metabolism, disease manifestation and progression between rodents and humans. Also, the differences in the absorption of purslane extract/powder in rodents and humans can limit the translatability of findings from rodents to humans. All these differences in mechanisms in rodents and humans could have caused the contrasting findings found in this study in relation to the effect of purslane on inflammatory markers.

Despite these limitations, we observed the positive effects of purslane in the markers of inflammation and oxidative stress under different doses, especially in rodent studies. This makes us hopeful for any future clinical studies. The preclinical studies used STZ, alloxan monohydrate and HDF feeding to induce diabetes; therefore, we can have confidence that the positive results were not due to the method used. The use of three independent researchers helped eliminate any bias in the search, selection and data extraction.

## 14. Conclusions

The evidence in this study supports the use of purslane as an antioxidant and anti-inflammatory agent in diabetes. While sufficient evidence was gathered from the rodent model of diabetes, the evidence from clinical trials also supports the potential benefits of purslane in ameliorating inflammation among T2D patients. The evidence for the effect of purslane on oxidative stress in clinical trials is limited and thus calls for future focus in clinical trials in this area. Although not every study focused on the same markers for these parameters, the evidence gathered in this review, especially the preclinical studies, suggests that purslane can alleviate inflammation and oxidative stress in T2D.

## 15. Future Recommendations

This review proves that purslane may be a suitable supplement and an alternative treatment to manage T2D. The lack of clinical trials that assess the effect of purslane in T2D on inflammation and oxidative stress calls for further clinical studies to be conducted, especially in countries where the prevalence of T2D is high. Such trials should employ sound methodology, use sufficient sample size, and further standardise effective doses of purslane and duration of intervention that can ameliorate inflammation and oxidative stress among T2D.

## Figures and Tables

**Table 1:** Search strategy adapted to the databases.

Database	Exact Search	Retrieved Records
PubMed	((portulaca oleracea[MeSH Terms]) OR (purslane[MeSH Terms])) AND (type 2 diabetes[MeSH Terms])	6
Scopus	-ABS-KEY (purslane) OR TITLE-ABS-KEY (portulaca AND oleracea) AND TITLE-ABS-KEY (type 2 diabetes AND mellitus) OR TITLEABS-KEY (type 2 diabetes)) AND (LIMIT-TO (DOCTYPE, “ar”))	24

**Table 2:** General overview of the effect of purslane extract on inflammation and oxidative stress in rodent models of diabetes.

Authors & Reference	Country	Experimental Model	Intervention and Duration	Summary of Findings
Hassan et al. [33]	Egypt	STZ-induced diabetes in adult male Sprague-Dawley rats.	Treated with 100 mg/kg ethanolic extract of purslane for 4 weeks.	TBARS level was decreased after treatment with purslane. Hippocampal GSH and IL-10 levels were increased after treatment with purslane. Reduced levels of TNF-α after treatment with purslane.
Sharma et al. [34]	India	STZ-induced diabetes in Sprague-Dawley rats and albino mice.	Treated with 100 and 250 mg/kg ethanolic extract of purslane for 3 weeks.	Treatment with purslane returned the activity of enzymatic antioxidants SOD and CAT to normal. Treatment decreased TBARS and increased GSH in the liver and kidney.
Samarghandian et al. [35]	Iran	STZ-induced Wistar albino rats.	Treated with either 100, 200 or 400 mg/kg aqueous extract of purslane for 4 weeks.	400 mg of purslane decreased serum IL-6 and MDA and increased GSH and TAC. 200 mg of purslane reduced serum levels of TNF-α and increased GSH.
Miao et al. [36]	China	HFD induced Male C57BL/6J mice and Sprague Dawley rats.	Treated with 400 mg/kg ethanolic extract of purslane for 4 weeks.	Purslane extract hindered high ROS levels in the aorta.
Ramadan et al. [37]	Egypt	Alloxan monohydrate induced diabetes in Wistar rats.	Treated with 250 mg/kg aqueous extract of purslane for 4 weeks.	Purslane extract reduced TNF-α and IL-6 levels.
Taha Mohammed et al. [22]	Iraq	STZ-induced diabetes in Wistar albino rats.	Treated with 200 mg/kg of aqueous extract of purslane for 3 weeks.	Purslane extract increased the activity of enzymatic antioxidants SOD and CAT.
Lee et al. [38]	Republic of Korea	Male C57BL/KsJ-db/db mice and wild-type C57BL/6J mice.	Treated with 300 mg/kg of aqueous extract of purslane for 10 weeks.	Purslane extract reduced concentration levels of NF-κβ p65. NF-κβ-DNA binding in renal nuclear extracts was reduced after treatment.
Sharma et al. [39]	India	STZ-induced diabetes in Sprague Dawley rats.	Treated with 25 and 50 mg/kg ethanolic extract of purslane polysaccharide fraction (PPF) for 3 weeks.	PPF decreased tissue TBARS and increased tissue GSH and GPx activity. PPFT increased the activity of enzymatic antioxidants CAT and SOD.
Saad et al. [40]	Egypt	STZ-induced diabetes in Wistar rats.	Treated with 200 mg/kg ethanolic extract of purslane for 30 days.	Purslane decreased MDA and increased GSH, TAC, SOD, and CAT activity. Reduced CRP, IL-6, and TNF-α levels.
Oyabambi et al. [41]	Nigeria	STZ-induced diabetes in Wistar rats.	Treated with 400 mg/k ethanolic extract of purslane for 4 weeks.	Purslane decreased TNF-α and IL-6 and increased GSH levels.

TBARS: thiobarbituric acid reactive substances; GSH: glutathione; SOD: superoxide dismutase, CAT: catalase, NF-κβ: nuclear factor kappa-β, MDA: malondialdehyde; GPx: Glutathione peroxidase, TAC: total antioxidant capacity.

**Table 3:** General overview of the effect of purslane extract on inflammation and oxidative stress in diabetic patients.

Author	Study Design	Country	Population	Age (Years)	BMI	Intervention and Duration	Summary of Findings
Zakizadeh et al. [43]	Cross-over randomised controlled clinical trial	Iran	40 T2D patients.	35–65	Not reported	10 g of purslane seed powder daily with 240 cc low-fat yoghurt for 5 weeks.	Purslane showed no significant effect on plasma TAC and MDA levels after treatment.
Dehghan et al. [42]	Double-blind study	Iran	196 T2D patients.	52.08 ± 3.45	29.5 ± 6.5	Patients consumed 2.5 g of purslane seed powder with lunch and 5 g with dinner for 16 weeks.	The protein and mRNA concentration levels of NF-κβ and CRP decreased after 16 weeks of treatment.

BMI: body mass index; T2D: type 2 diabetes; TAC: total antioxidant capacity; MDA: malonaldehyde; mRNA: messenger ribosomal nucleic acid; NF-κβ: nuclear factor kappa-β; CRP: C-reactive protein.

## Data Availability

Not applicable.

## Supplementary Materials

The following supporting information can be downloaded at: https://www.mdpi.com/article/10.3390/ijms252212276/s1.
